# Distal cis-regulatory elements in 3′ UTRs orchestrate gene expression via single-gene domain architecture in *Brassica rapa*

**DOI:** 10.1093/hr/uhag236

**Published:** 2026-06-10

**Authors:** Lupeng Zhang, Xu Cai, Yihan Xu, Jun Shi, Jian Wu, Xiaowu Wang

**Affiliations:** State Key Laboratory of Vegetable Biobreeding, Institute of Vegetables and Flowers, Chinese Academy of Agricultural Sciences, Beijing 100081, China; State Key Laboratory of Vegetable Biobreeding, Institute of Vegetables and Flowers, Chinese Academy of Agricultural Sciences, Beijing 100081, China; State Key Laboratory of Vegetable Biobreeding, Institute of Vegetables and Flowers, Chinese Academy of Agricultural Sciences, Beijing 100081, China; State Key Laboratory of Vegetable Biobreeding, Institute of Vegetables and Flowers, Chinese Academy of Agricultural Sciences, Beijing 100081, China; State Key Laboratory of Vegetable Biobreeding, Institute of Vegetables and Flowers, Chinese Academy of Agricultural Sciences, Beijing 100081, China; State Key Laboratory of Vegetable Biobreeding, Institute of Vegetables and Flowers, Chinese Academy of Agricultural Sciences, Beijing 100081, China

## Abstract

Cis-regulatory elements (CREs) are fundamental to precise gene regulation. Although distal CREs located in intergenic regions have been extensively studied, the functional landscape of those residing in 3′ untranslated regions (UTRs) remains largely unexplored. In this study, we generate comprehensive epigenomic profiles of *Brassica rapa* leaves at two developmental stages (rosette and reproductive), integrating ATAC-seq, CUT&Tag for five histone modifications (H3K4me3, H3K27ac, H3K27me3, H3K36me3, H3K9me2), and BL-Hi-C data. We identify 6362 CREs within 3′ UTRs, defined as accessible chromatin regions within 0.5 Kb of transcription termination sites. These CREs within 3′ UTRs are functionally classified by histone marks: H3K27ac-marked, H3K27me3-marked, and bivalent CREs. Using BL-Hi-C at resolutions up to 100 bp, we uncover that these CREs within 3′ UTRs contribute to the formation of single-gene domains. Comparative analysis between developmental stages reveals that these distal CREs orchestrate stage specific gene expression by modulating chromatin interaction, a mechanism exemplified by the flowering regulator *BrFLC2*. The transcriptional activation activity of the distal CRE of *BrFLC2* was verified by dual-luciferase reporter assays. Together, our findings establish 3′ UTR embedded distal CREs as critical regulatory elements that shape gene expression through single-gene domain architecture in *B. rapa*.

## Introduction

CREs are fundamental genomic regions that orchestrate precise spatiotemporal patterns of gene expression [1]. In plants, comprehensive epigenomic mapping has enabled genome-wide identification of such elements [2], which are broadly categorized based on their location relative to genes: proximal CREs typically reside near transcription start sites (TSS), while distal CREs are located in intergenic regions or within UTRs [3]. Although the regulatory roles of intergenic distal CREs have been increasingly recognized for instance in modulating maize *tb1* expression [4], fine-tuning specialized metabolism in tomato [5], and governing nitrogen utilization in rice [6], the functional landscape and regulatory mechanisms of distal CREs residing in 3′ UTRs remain largely unexplored.

Previous studies have established that epigenetic mechanisms are integral to agronomic traits and environmental adaptation in plants [7]. Key examples include H3K27me3 mediated repression of *FLC* to promote flowering [8]. In wheat, histone modifications dynamically regulate the expression of key genes during vernalization [9]. In tomato, 3D chromatin structure is involved in the heat stress response [10]. In rice, circadian oscillations of H3K9ac histone modification regulate the expression of circadian rhythm-related genes [11]. *Brassica rapa* (*B. rapa*) is a globally important vegetable crop with substantial economic value and also serves as an excellent model for evolutionary epigenomics. Studies in *B. rapa* have revealed the epigenome’s role in maintaining subgenome transcriptional balance in allotetraploid *B. napus* [12], as well as epigenome dynamic changes following whole-genome duplication [13].

In eukaryotes, chromatin is folded into topologically associating domains (TADs), which are self-interacting regions with insulated boundaries, as revealed by Hi-C contact maps [14]. Dynamic changes in these higher-order structures have been linked to key agronomic traits. For example, TAD dynamics influence fiber length in cotton [15], and the emergence of a new TAD has been associated with dwarfism in peanut [16]. However, in plants, the absence of CTCF, a key architectural protein in animals [17] suggests that TADs represent a more plastic and less canonical level of organization. Instead, plant genomes are organized into various TAD-like structures, including intergenic condensed spacers (ICONS) [18] and compartment domains [19]. More recently, the application of high-resolution techniques such as Micro-C has revealed even finer structural units termed single-gene domains in *Arabidopsis* and rice [20, 21].

As a critical component of the epigenome, 3D chromatin architecture has also been investigated in *Brassica*, revealing conserved features of compartments and TADs [22], as well as their dynamic response to heat stress [23]. However, these studies have relied on conventional Hi-C, which typically achieves resolutions of 2-5 Kb, sufficient for identifying large-scale structural features such as compartments and TADs, but inadequate for resolving finer structural units such as single-gene domains or for dissecting the chromatin interactions of individual genes. Consequently, whether distal CREs contribute to the formation of single-gene domains in *B. rapa*, and whether such structures undergo reorganization during development, remain unknown.

In this study, we generated comprehensive epigenetic profiles from *B. rapa* leaves at both rosette and reproductive stages, encompassing five histone modifications (H3K4me3, H3K27ac, H3K27me3, H3K36me3 and H3K9me2), chromatin accessibility (ATAC-seq) and chromatin conformation (BL-Hi-C). By integrating these multi-omics datasets, we identified thousands of distal CREs specifically within 3′ UTRs and revealed that they are prominently marked by either H3K27ac or H3K27me3. Leveraging BL-Hi-C at resolutions up to 100 bp, we uncovered that these distal CREs frequently participate in the formation of single-gene domains. Comparative analysis between developmental stages further demonstrated that distal CREs orchestrate stage specific gene expression through the dynamic modulation of single-gene domain architecture, as exemplified by the flowering regulator *BrFLC2*. Collectively, this work provides a rich epigenetic resource for *B. rapa* research and reveals a previously underappreciated mechanism by which 3′ UTR embedded distal CREs regulate gene expression via single-gene domain architecture.

## Results

### Epigenome profiling of *B. rapa* leaves

We selected rosette and reproductive stages of *B. rapa* to investigate epigenetic features (Fig. S1). A total of 12 genome-wide epigenetic datasets were generated, encompassing profiles of five histone modifications (H3K4me3, H3K27ac, H3K27me3, H3K36me3, and H3K9me2), chromatin accessibility, and transcriptomes, each with two biological replicates from both developmental stages (Tables S1 and S2). All replicates exhibited high reproducibility, with Pearson correlation coefficients exceeding 0.97 (Fig. S2). Data quality was assessed via inter-replicate correlation analysis and the fraction of reads in peaks (FRiP). FRiP values for histone modification datasets were ~ 0.6, while those for chromatin accessibility data exceeded 0.3 (Table S3).

As expected, the constitutive heterochromatin mark H3K9me2 was highly concentrated in pericentromeric regions. In contrast, the facultative heterochromatin mark H3K27me3 was enriched in gene-dense telomeric arms. Euchromatin marks H3K4me3, H3K27ac, and H3K36me3, together with ATAC-seq signals, were also enriched in telomeric regions (Fig. 1A). We examined the distribution of these chromatin features relative to genes. Referring to the classification in other species [24, 25], proximal regions were defined as 0.5 Kb upstream and downstream of TSS; distal regions were defined as 0.5 Kb upstream and downstream of transcriptional terminal sites (TTS); the region between 0.5 Kb downstream of TSS and 0.5 Kb upstream of TTS was defined as the genic region, and remaining portions of the genome were classified as intergenic regions. The histone modifications H3K4me3, H3K27ac, H3K27me3 and H3K36me3, along with accessible chromatin, are located in the distal region at rates of 16%, 20.8%, 15.3%, 14.4%, and 18.6%, respectively, indicating the presence of extensive CREs in the distal region (Fig. 1B).

**Figure 1:**
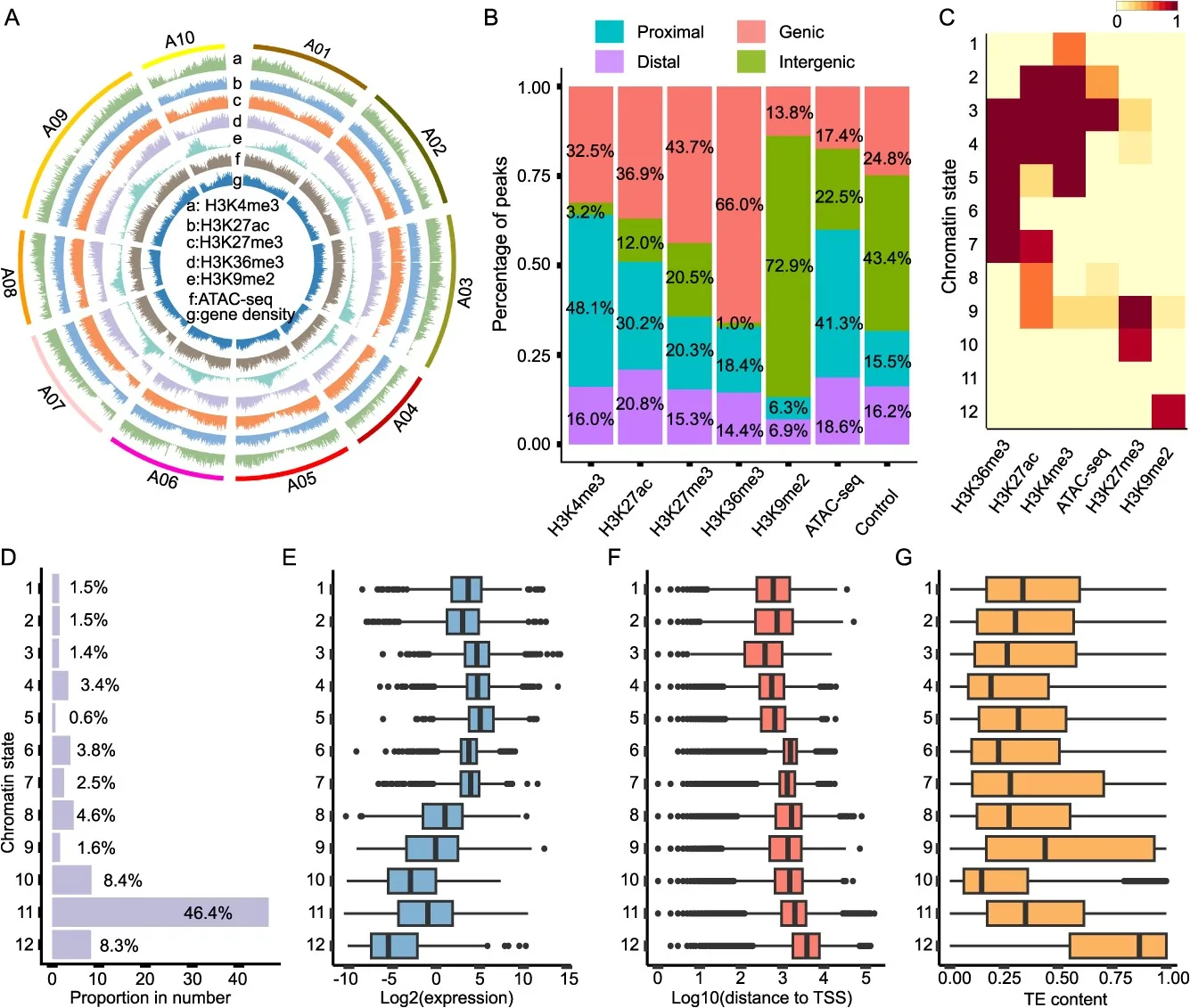
Epigenome profiling of *B. rapa* leaf at rosette stage. (A) Chromosomal landscape of genomic features and chromatin modifications. Rings represent the enrichment of H3K4me3 (a); the enrichment of H3K27ac (b); the enrichment of H3K27me3 (c); the enrichment of H3K36me3 (d); the enrichment of H3K9me2 (e); the enrichment of ATAC-seq (f); and gene density (g). (B) Distribution of epigenetic features across genomic regions including proximal, distal, genic, and intergenic. Randomly selected 20,000 genomic regions were used as controls. (C) Chromatin states classified according to the epigenetic features using a multivariate HMM. In figures C-G, numbers 1–12 represent the 12 chromatin states. (D) The proportion of each chromatin state in the whole genome. (E) Gene expression levels associated with each chromatin state. (F) Distance of each chromatin state to the nearest TSS. (G) TE content within each chromatin state.

To systematically analyze CREs, we applied a multivariate Hidden Markov Model (HMM) that integrates combinatorial epigenetic signals and partitioned the genome into 12 functionally distinct chromatin states (Fig. 1C). States associated with promoters (States 1-5) showed high frequencies of H3K4me3, covered 8.4% of the genome (Fig. 1D), and were largely depleted of transposable elements (TEs) (Fig. 1G). States 6-10, located distal to TSS, represent potential distal elements (Fig. 1F). Among these, States 6 and 7 were enriched for H3K36me3 and corresponded to gene bodies with putative regulatory roles. State 8 was defined by a predominant H3K27ac signal, designating it as a distal transcriptional activation element. Conversely, State 10 was primarily marked by H3K27me3, representing a distal transcriptional repressive elements. State 9 exhibited enrichment for both H3K27ac and H3K27me3, indicative of a bivalent chromatin state poised for regulatory transitions. State 11 encompassed unannotated chromatin regions, while State 12, marked exclusively by H3K9me2, corresponded to transcriptionally inactive heterochromatin. These chromatin states displayed distinct modification and accessibility profiles and were associated with varying expression levels of their corresponding genes (Fig. 1E). Comparative analysis between the two developmental stages revealed that histone modification patterns were largely stable, whereas chromatin accessibility exhibited pronounced dynamics. For instance, accessibility decreased in State 3 but increased in State 5 during the reproductive stage, suggesting stage specific regulatory rewiring (Fig. S5).

### Identification and characterization of the distal CREs in *B. rapa*

Based on ATAC-seq data from *B. rapa* rosettes, we identified 14 498 proximal and 6362 distal CREs. Considering that accessible chromatin regions frequently coincide with transcription factor (TF) binding sites, we performed de novo motif analysis on both CRE sets using Homer. The results indicate that proximal and distal CREs sequence are enriched for similar motifs. The five most significantly enriched motifs were PCF, RAMOSA, ABI5, and TF3A, and Os05g0497200 in proximal CREs and CRF10 in distal CREs (Fig. 2A). These findings demonstrate that distal CREs are regulated by TFs similar to those acting on proximal CREs.

**Figure 2:**
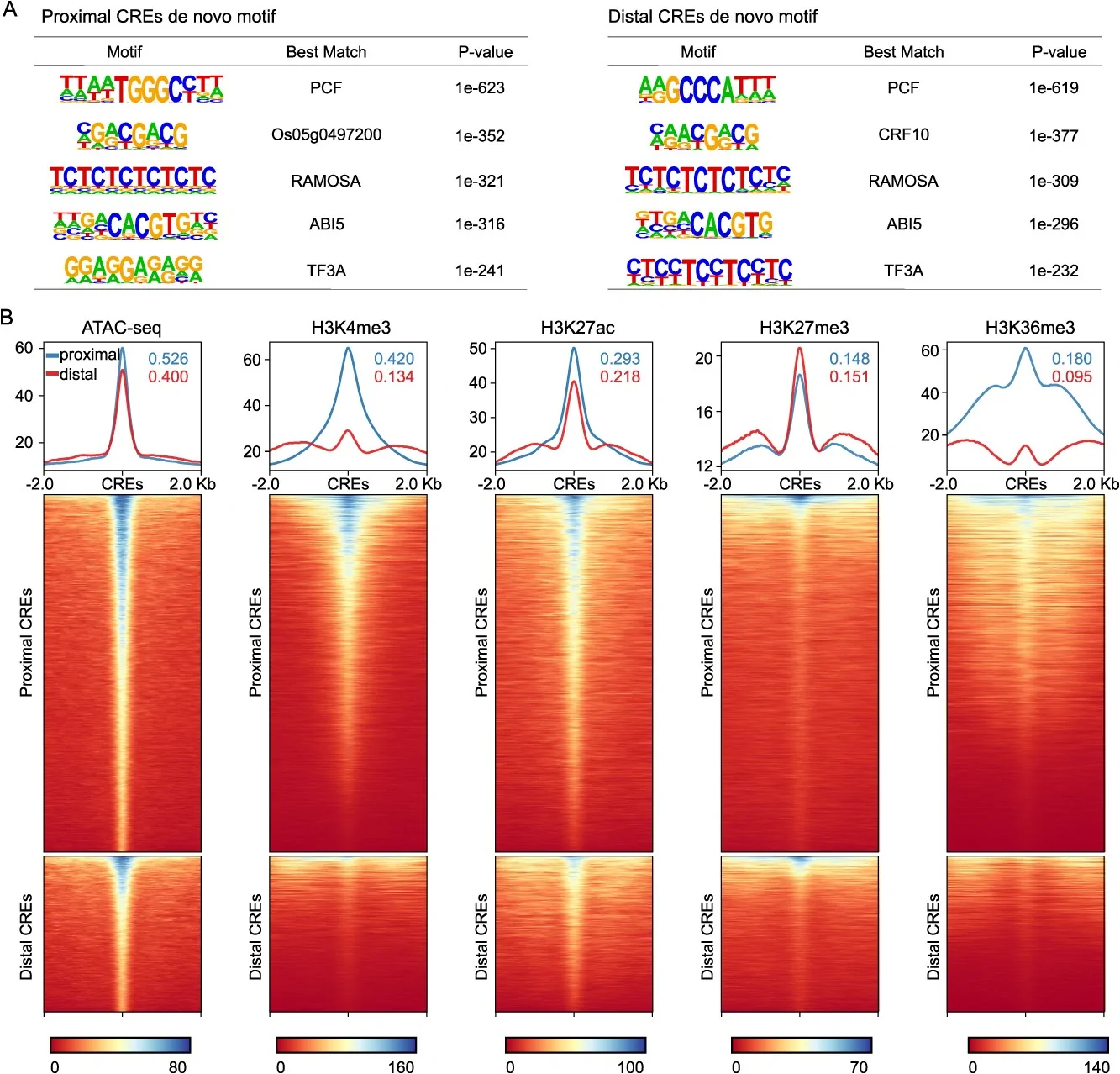
Identification and characterization of distal CREs in *B. rapa*. (A) The top five enriched TFs and their corresponding binding motifs in proximal CREs (left) and distal CREs (right) relative to genomic background. (B) Chromatin accessibility and epigenetic modification profiles across proximal and distal CREs. Profiles include transcriptionally active marks (H3K4me3, H3K27ac, H3K36me3) and repressive mark (H3K27me3) within ±2 kb genomic regions centered on CREs. The enrichment score (top-right) was calculated as the signal intensity in the central 100 bp of CREs divided by the signal intensity in the flanking 500 bp regions.

We next compared the epigenetic modification profiles between the two CRE categories. Chromatin accessibility was slightly higher in proximal CREs than in distal CREs, with enrichment scores of 0.526 vs. 0.400. Histone modification analysis further revealed that the transcriptionally active marks H3K4me3 and H3K36me3 were markedly enriched in proximal CREs (enrichment scores: 0.42 and 0.18), whereas their levels in distal CREs were much lower (0.134 and 0.095). In contrast, the transcriptional active mark H3K27ac exhibited comparable enrichment scores in proximal and distal CREs (0.293 for proximal CREs and 0.218 for distal CREs), as well as the transcriptionally silencing mark H3K27me3 (0.151 for proximal CREs and 0.148 distal CREs) (Fig. 2B). These results suggest that proximal CREs primarily function as promoters, whereas distal CREs putatively operate as either enhancers or silencers.

### Functional classification of distal CREs

By integrating H3K27ac and H3K27me3 histone modification data, we classified distal CREs of rosette stage into distinct functional categories: 1929 CREs marked by H3K27ac, 609 CREs marked by H3K27me3 and 2256 bivalent CREs (Tables S4 and S5). The bivalent CREs, characterized by the co-presence of H3K27ac and H3K27me3 marks, reflect a plastic chromatin state (Fig. 3A). Comparative analysis of chromatin accessibility across these three states revealed that H3K27ac-marked CREs and H3K27me3-marked CREs exhibit similar levels of chromatin accessibility, while bivalent CREs display reduced chromatin accessibility (Fig. 3B). Gene expression analysis associated with each chromatin state showed that genes linked to H3K27ac-marked CREs were expressed at significantly higher levels than those associated with bivalent states (*P* = 4.6 × 10^-6^). In contrast, genes linked to H3K27me3-marked CREs showed significantly lower expression compared to bivalent states (*P* < 2.2 × 10^-16^) (Fig. 3C). These results demonstrate that distal CREs can substantially influence the transcription process.

**Figure 3:**
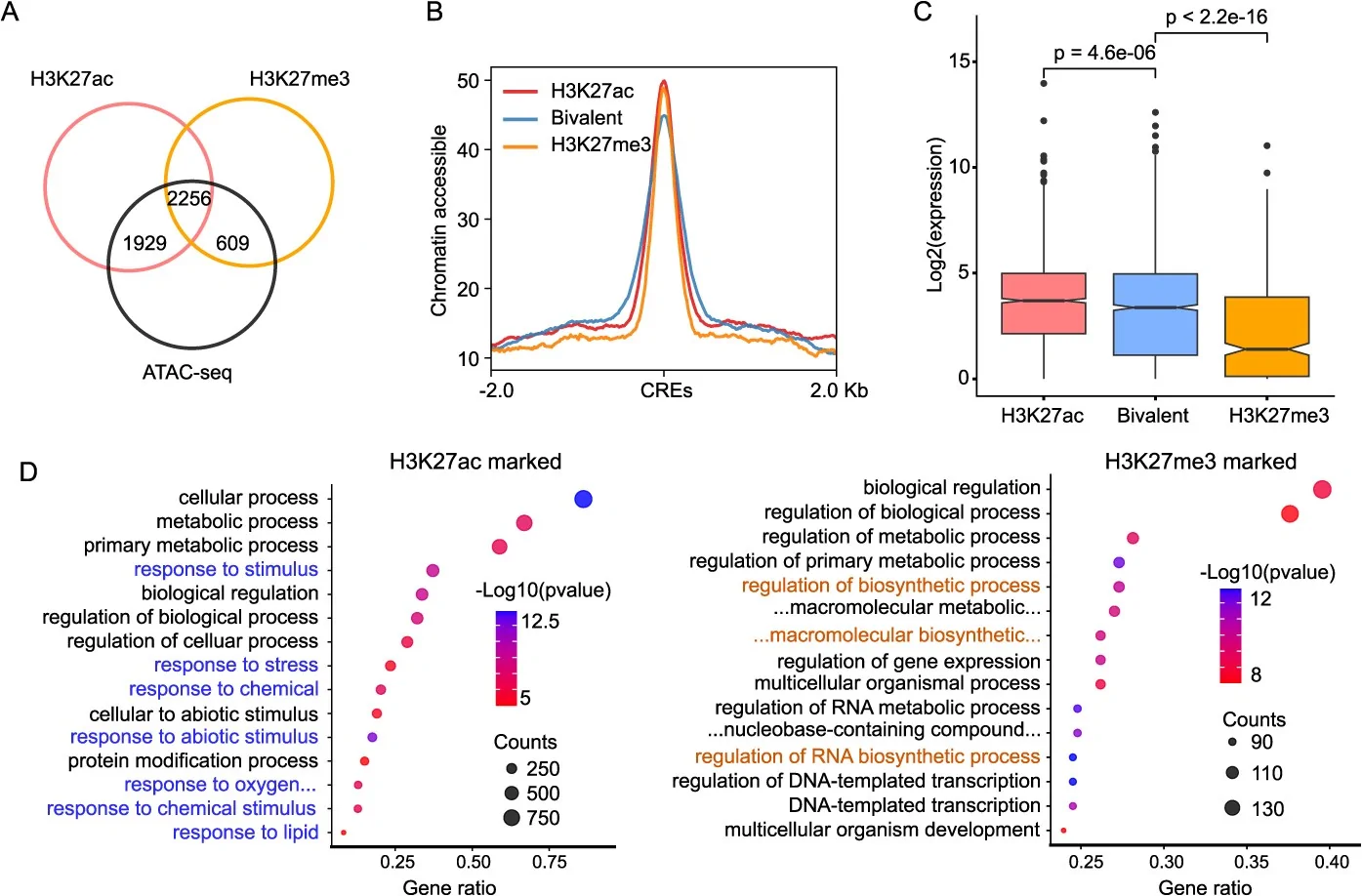
Functional classification of distal CREs. (A) Overlap of H3K27ac and H3K27me3 signals with distal CREs. Regions carrying both marks were designated as bivalent CREs. (B) Comparison of chromatin accessibility across H3K27ac-marked, H3K27me3-marked and bivalent CREs. (C) Impact of distal H3K27ac, H3K27me3, and bivalent CREs on gene expression levels. (D) GO enrichment analysis of genes linked to distal H3K27ac and H3K27me3.

To gain insight into the biological processes potentially regulated by these CREs, we performed Gene Ontology (GO) enrichment analysis on genes associated with H3K27ac-marked and H3K27me3-marked CREs. Both categories were enriched in broad regulatory terms such as ‘metabolic process’ and ‘biological regulation’. H3K27ac-marked genes were specifically enriched in stimulus responsive pathways including ‘response to stimulus’ and ‘response to stress’ (Fig. 3D). This suggests that enhancer-regulated genes may be poised for rapid transcriptional activation in response to environmental or cellular cues. In contrast, H3K27me3-marked genes were uniquely enriched in biosynthetic regulatory pathways, such as ‘regulation of biosynthetic process’ and ‘regulation of macromolecule biosynthetic process’, which are typically associated with more stable, constitutive expression programs (Fig. 3D). Together, these findings indicate a functional specialization between H3K27ac-marked and H3K27me3-marked gene sets. The H3K27ac-marked genes predominantly participating in stimulus responsive programs and H3K27me3-marked genes modulating biosynthetic processes.

### Fine-scale 3D structure reveals distal CREs involved in single-gene domain formation

To investigate the role of distal CREs in higher-order chromatin architecture, we performed BL-Hi-C on *B. rapa* leaves from both rosette and reproductive stages, generating ~210 Gb of raw sequencing data. After alignment and filtering (MAPQ > 10), we obtained ~290 million valid contacts (Tables S1 and S2). The ratio of intrachromosomal vs. interchromosomal contacts, which is generally considered to represent the signal-to-noise ratio, in our BL-Hi-C data were 4.4 ± 1, indicating that our BL-Hi-C data are of high quality (Tables S1 and S2). Given the strong reproducibility between replicates (Pearson correlation > 0.98) (Fig. S3), we merged replicate datasets for downstream analyses. To benchmark our method, we compared BL-Hi-C with conventional Hi-C across a random chromatin region at varying resolutions (500, 200 and 100 bp) (Fig. 4). BL-Hi-C consistently yielded stronger and finer chromatin contact signals with lower background noise. At 500 bp resolution, BL-Hi-C detected obvious chromatin loops that were not discernible by Hi-C. At 200 bp resolution, BL-Hi-C revealed stronger single-gene domain structures compared to Hi-C. BL-Hi-C could even resolve clear single-gene domains at resolutions as high as 100 bp, whereas Hi-C failed to detect such structures (Fig. 4). Consistent single-gene domains were observed across two biological replicates (Fig. S4), further confirming the high quality of our BL-Hi-C data.

**Figure 4:**
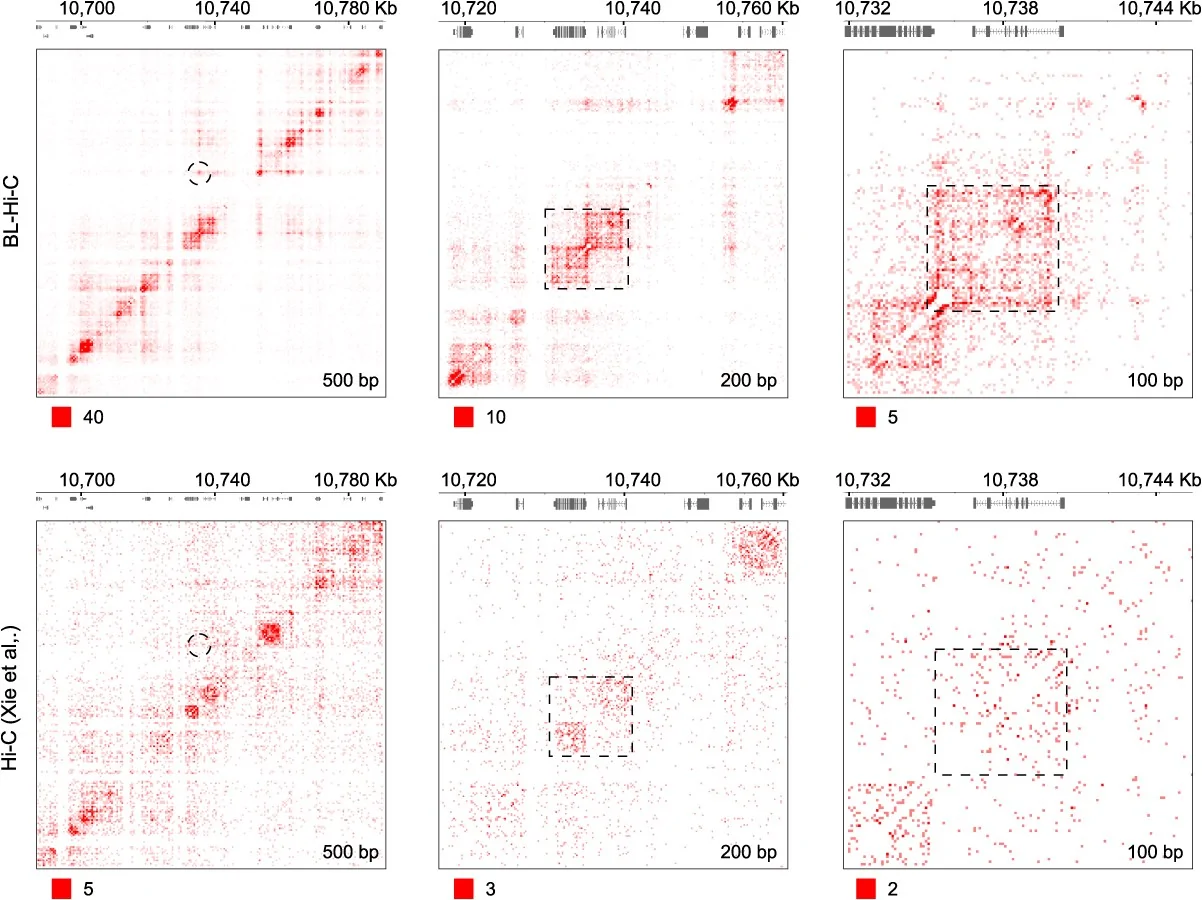
Contact matrix maps of BL-Hi-C (upper panel) and Hi-C [22] (bottom panel) at various resolutions of the region of chromosome A02. The circles indicate chromatin loops, and the squares indicate domains in the heatmaps. The numbers below indicate the maximum values in the heatmap.

Leveraging this interaction map, we investigated whether distal CREs contribute to the formation of single-gene domains. According to the presence or absence of accessible chromatin regions at gene boundaries, we classified genes into four categories: those with accessible chromatin regions at both TSS and TTS (Both), those without accessible chromatin regions at either end (None), those with accessible chromatin regions only at TSS (TSS only), and those with accessible chromatin regions only at TTS (TTS only). Visualization of pile-up contact matrices revealed that genes with accessible CREs at either end (Both, TSS only or TTS only) exhibited stronger single-gene domain structures compared to those lacking accessible CREs at both ends (None) (Fig. 5A). To quantify these differences, we calculated intragenic interaction strength as the average interaction frequency among all bin pairs located within the TSS to TTS interval. The average intragenic interaction strengths for Both, TSS only, TTS only and None genes were 653, 729, 601, and 321, respectively (Fig. 5A). These results demonstrate that the presence of accessible CREs at gene boundaries is associated with enhanced single-gene domain structures.

**Figure 5:**
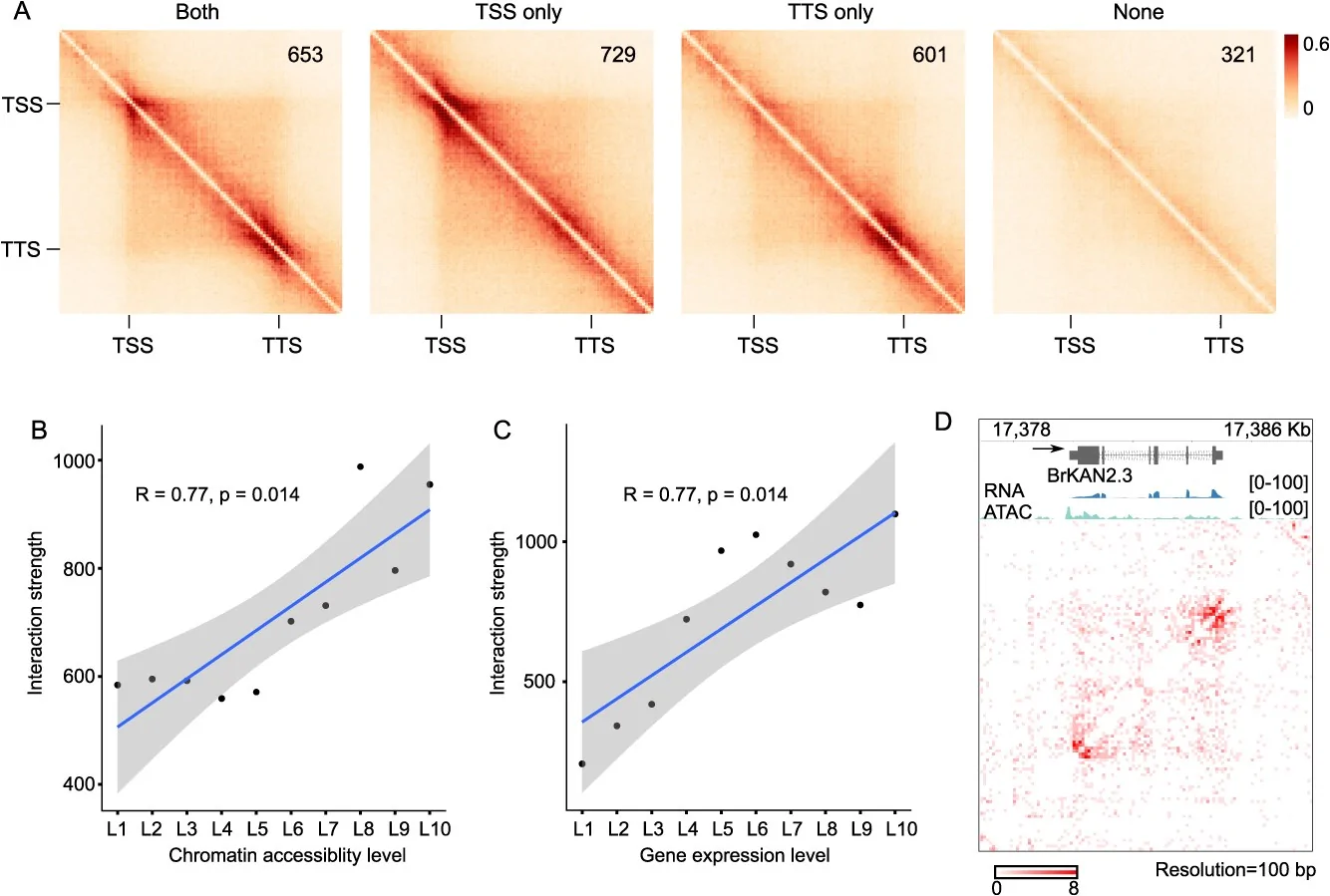
Distal CREs form single-gene domain structures to regulate gene expression. (A) Pile-up images of BL-Hi-C contact matrices for *B. rapa* genes grouped by chromatin accessibility patterns at gene boundaries. Genes were classified into four categories: those with accessible chromatin regions at both TSS and TTS (Both), those with accessible chromatin regions only at the TSS (TSS only), those with accessible chromatin regions only at the TTS (TTS only), and those with accessible chromatin regions at neither end (None). Contact matrices were aligned by gene bodies and rescaled to equal dimensions for visualization. TSS and TTS positions are indicated. (B) Scatter plot showing the correlation between chromatin accessibility of distal CREs and intragenic interaction strength. Genes were grouped into 10 quantiles based on distal CRE accessibility levels. Spearman’s *R* = 0.77, *P* = 0.014 (*n* = 10 quantiles). (C) Scatter plot showing the correlation between gene expression levels and intragenic interaction strength. Genes were grouped into 10 quantiles based on expression levels. Spearman’s *R* = 0.77, *P* = 0.014 (*n* = 10 quantiles). (D) The adaxial–abaxial polarity related gene *BrKAN2.3* (*BraA05g024090.3.5C*) forms a single-gene domain. The black arrow indicates the gene orientation.

To further explore the relationship between the chromatin accessibility of CREs and domain strength, we stratified distal CREs into 10 equally sized bins (L1-L10) based on their chromatin accessibility levels and calculated the corresponding intragenic interaction strength for each bin (Fig. 5B). Intragenic interaction strength increased progressively with higher chromatin accessibility (Spearman’s *R* = 0.77, *P* = 0.014), indicating a positive relationship between CRE accessibility and domain organization. Similarly, intragenic interaction strength correlated positively with gene expression levels (Spearman’s *R* = 0.77, *P* = 0.014), suggesting that stronger single-gene domain structures are associated with more active transcriptional states (Fig. 5C).

*KAN* family genes play a key role in leaf morphogenesis by regulating adaxial-abaxial polarity [26]. As an example, we examined *BrKAN2.3*, which resides within a distinct single-gene domain delineated by its upstream and downstream regions, with domain boundaries coinciding with accessible chromatin regions (Fig. 5D). This domain structure was maintained during the reproductive stage, and a similar organization was observed for its homolog *BrKAN2.1*. The expression of two *BrKAN2* was also stable during both periods (Fig. S6). Collectively, these results indicate that distal CREs particularly those marked by accessible chromatin at gene boundaries play an active role in establishing and maintaining single-gene domain architecture in *B. rapa*.

### Distal CREs regulate the gene expression through single-gene domains

Comparative analysis of distal accessible chromatin regions (dACRs) between the rosette and reproductive stages identified 1447 enhanced and 2195 reduced dACRs, using a fold-change threshold of >2. By assessing expression changes based on the ratio of gene expression between these two stages, we observed that genes associated with enhanced dACRs exhibited significantly higher fold change of expression compared to those linked to reduced dACRs (*P* < 2.2 × 10^-16^). This suggests that changes in dACRs influence gene expression (Fig. 6A). Applying the same approach, we evaluated the effect of altered dACRs on histone modifications. The results demonstrated that active histone marks H3K4me3, H3K27ac, and H3K36me3 were significantly enriched in enhanced dACRs relative to reduced dACRs. In contrast, the repressive mark H3K27me3 was more abundant in reduced dACRs (Fig. 6A). These findings indicate that histone modification patterns correspond to dACR changes and collectively contribute to gene regulation. However, the participation of other epigenetic modifications, such as DNA methylation, RNA methylation, and noncoding RNAs, on the regulation of gene expression cannot be ruled out.

**Figure 6:**
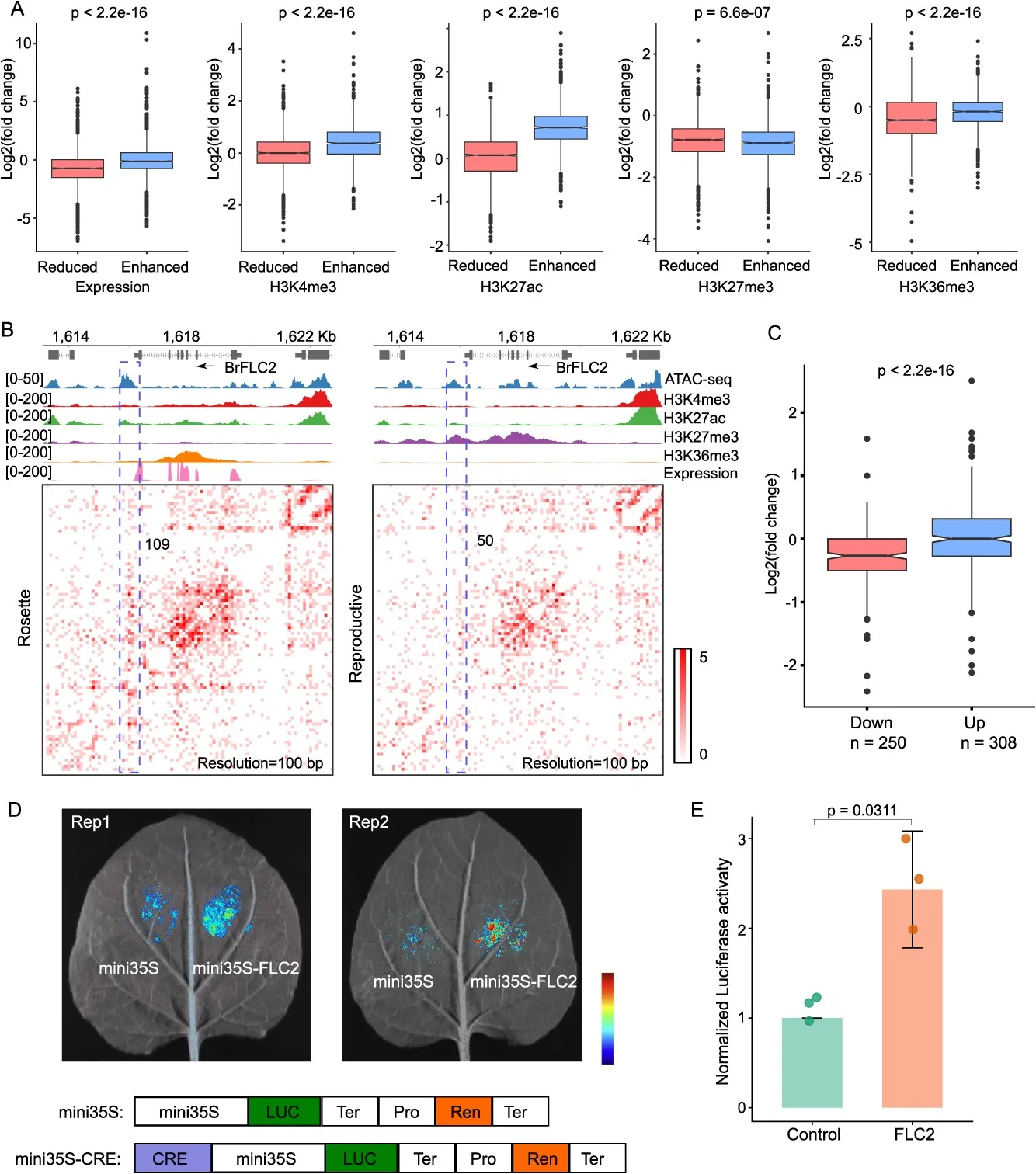
Distal CREs regulate the gene expression through single-gene domains. (A) Changes in expression and histone modification levels (H3K4me3, H3K27ac, H3K27me3 and H3K36me3) in enhanced and reduced dACRs. The fold change is calculated as the ratio of the reproductive stage to the rosette stage. *P* values were calculated by performing a two-sided Wilcoxon test. (B) Reduced interactions of distal CRE at the *BrFLC2* (*BraA02g003370.3.5C*) locus, with a concordant decrease in its expression, a reduction in active histone marks, and an enrichment of repressive histone modifications. (C) Boxplot comparing the changes in interaction frequency between up-regulated and down-regulated genes, counting only interactions involving distal CREs. The fold change is calculated as the ratio of the reproductive stage to the rosette stage. *P* values were calculated by performing a two-sided Wilcoxon test. (D) Functional validation of distal CREs associated with *BrFLC2* via dual-luciferase reporter assay. The transcriptional regulatory activities of the distal CREs were evaluated using a transient expression system in *N. benthamiana* leaves. (E) Normalized luciferase activity was calculated using the ratio of Firefly luciferase (LUC) and Renilla luciferase (REN), and the LUC/REN ratio is presented as mean ± SD from three biological replicates. Statistical significance was determined using Student’s *t*-test (compared to the empty control).

We used *BrFLC2*, a key flowering gene [27], as an example to illustrate how distal CREs regulate gene expression. During the rosette stage, the distal CREs of *BrFLC2* exhibited robust chromatin accessibility. The gene body was enriched for active marks (H3K4me3, H3K27ac, H3K36me3) and showed relatively low levels of the repressive mark H3K27me3, consistent with high gene expression. In contrast, during the reproductive stage, these active modifications were reduced, accompanied by a marked increase in H3K27me3 enrichment. Concomitantly, BL-Hi-C analysis revealed that *BrFLC2* formed a distinct single-gene domain spanning from TSS to TTS during the rosette stage. This domain structure was weakened in reproductive stage, with the number of interactions involving distal CREs decreasing from 109 to 50 (Fig. 6B). These results suggest that the distal CRE of *BrFLC2* acts as a transcriptional activation element to regulate gene expression through chromatin interaction. Consistent with this pattern, similar developmental dynamics were observed for the *BrFLC2* homologs *BrFLC1* and *BrFLC3* (Fig. S7).

Subsequently, we performed luciferase reporter assays to validate the transcriptional activation activity of the distal CRE of *BrFLC2*. The candidate was cloned into the pGreenII-0800-miniLUC reporter vector and transiently expressed in *Nicotiana benthamiana* leaves, with a construct containing only the mini35S promoter serving as the control. Compared with the empty control, CRE containing constructs exhibited stronger fluorescence intensity (Fig. 6D). Consistently, measurement of LUC/REN ratios confirmed significantly higher activity for all tested CRE (with three biological replicates) (Fig. 6E), providing direct experimental evidence that the distal CRE can function as a transcriptional activation element.

To extend these observations beyond individual loci, we systematically examined the relationship between distal CRE interaction strength and gene expression at the genome-wide level. Through differential expression analysis, we obtained 1360 up-regulated and 1436 down-regulated genes. By quantifying the change in distal CRE interaction frequency between the two developmental stages, we found that up-regulated genes exhibited significantly stronger interaction strength compared to down-regulated genes (Fig. 6C). This genome-wide analysis supports the notion that distal CREs contribute to dynamic gene regulation through the modulation of single-gene domain architecture.

Collectively, these findings demonstrate that distal CREs and their associated single-gene domains undergo coordinated reprogramming during development, providing a structural framework for stage-specific gene expression in *B. rapa*.

## Discussion

ATAC-seq and DNase-seq are widely employed for genome-wide identification of potential CREs [24, 28]. In this study, we applied ATAC-seq to systematically map distal CREs in *B. rapa*, defining them as accessible chromatin regions within 0.5 Kb of TTS. This definition specifically targets elements residing in or near 3′ UTRs, a class of regulatory regions often overlooked in previous intergenic centric analyses [5, 29]. Through integration with histone modification profiles, we classified these distal CREs into H3K27ac-marked CREs and H3K27me3-marked CREs. These H3K27ac-marked and H3K27me3-marked CREs represent putative enhancers and silencers, respectively, but further experimental validation is required. GO enrichment analysis revealed that H3K27ac associated genes are enriched in stimulus responsive pathways, while H3K27me3 associated genes are enriched in biosynthetic regulatory pathways, suggesting functional specialization among these distal regulatory elements.

To investigate how these distal CREs function within higher-order chromatin architecture, we generated chromatin interaction maps using BL-Hi-C. Unlike animal genomes, many plants possess compact genomes with densely packed gene regions, necessitating resolutions of at least 1 Kb to resolve single-gene chromatin interactions. In *Arabidopsis*, the advanced Micro-C method achieves single-gene resolution but requires substantial sequencing depth (exceeding 1600-fold coverage) [21]. By contrast, our BL-Hi-C approach achieves comparable resolution (up to 100 bp) with ~260-fold coverage, largely due to its enhanced signal-to-noise ratio [30]. This technical advancement enabled us, for the first time in *B. rapa*, to systematically observe single-gene domains and to quantify their strength in relation to distal CRE accessibility and gene expression levels. These findings establish BL-Hi-C as an efficient tool for fine-scale chromatin architecture analysis in plant species with compact genomes.

While long-range enhancer-promoter interactions spanning over 100 Kb are well documented in animals [31–33], such long-distance regulatory contacts are rarely reported in plants. This discrepancy is likely attributable to the high gene density and compact genome architecture of many plant species [21], which often position enhancer-like elements within introns or UTRs rather than in distal intergenic regions, resulting in relatively shorter interaction distances, typically ranging from several kilobases to tens of kilobases in *Arabidopsis* [34–36]. In addition, single-gene domains formed by short-range interactions have been observed in *Arabidopsis* and rice using Micro-C [20]. Our study extends these findings by demonstrating that distal CREs located in 3′ UTRs actively participate in the formation of single-gene domains in *B. rapa*. Within such domains, these regulatory elements frequently interact with their promoters to establish local regulatory hubs, providing a structural framework for precise gene regulation. Our developmental stage comparison reveals that these single-gene domains are not static but undergo dynamic reorganization, suggesting that domain architecture may influence stage specific gene expression. This analysis provides a foundation for future investigations aimed at dissecting the specific chromatin loops that orchestrate these regulatory processes.

The developmental changes of single-gene domains are exemplified by the flowering regulator *BrFLC2*. During the rosette stage, *BrFLC2* forms a robust single-gene domain with strong intragenic interactions, accompanied by active histone modifications (H3K4me3, H3K27ac) and high expression levels. In the reproductive stage, this domain structure is weakened, coinciding with a reduction in active marks, enrichment of H3K27me3, and decreased expression. This coordinated reprogramming of chromatin architecture, along with histone modifications, regulates gene expression. Genome-wide analysis further supports this model, as up-regulated genes exhibit significantly stronger domain structures compared to down-regulated genes. Collectively, these findings indicate that distal CREs contribute to stage specific gene regulation by modulating single-gene domain architecture, adding a new dimension to our understanding of how plants coordinate developmental transitions.

Despite these advances, several limitations should be acknowledged. First, the materials used in this study were all young leaves, which precluded investigation of the epigenetic changes in shoot apical meristem-specific genes during vernalization. Second, while our dual-luciferase assays confirmed transcriptional activation activity of selected distal CRE in a heterologous system (*N. benthamiana*), validation in native *B. rapa* using genome editing approaches (e.g., CRISPR-Cas9) would provide more definitive evidence of its endogenous regulatory functions. Third, the molecular mechanisms underlying single-gene domain formation remain unclear; whether specific TFs, structural proteins (such as plant-specific analogs of CTCF), or transcriptional activity itself drives domain establishment warrants further investigation.

Future studies could address these questions by integrating BL-Hi-C with perturbations of candidate TFs or structural proteins to dissect the causal relationships between CRE accessibility, domain formation, and gene expression. Additionally, applying chromatin conformation capture to additional tissues and environmental conditions would reveal the extent to which single-gene domain changes contribute to phenotypic plasticity and environmental adaptation in *B. rapa* and related *Brassica* species.

## Conclusion

In conclusion, this study presents a comprehensive epigenomic landscape of *B. rapa*, identifies thousands of previously uncharacterized distal CREs within 3′ UTRs, and reveals their role in orchestrating gene expression through single-gene domain architecture. These findings establish 3′ UTR embedded distal CREs as critical regulatory elements and provide a valuable resource for future functional studies aimed at fine-tuning gene expression in *Brassica* crops.

## Material and methods

### Plant material and growth conditions

*B. rapa* (*L. ssp. pekinensis*, acc. Chiifu) seeds were germinated on Petri dishes at 25°C for 14 h, after which the seedlings were transferred to soil and grown in a greenhouse under a 16 h photoperiod at 23°C. Leaves were harvested from 40-day-old plants for the construction of rosette stage libraries. For the reproductive stage libraries, plants were first subjected to 30 days of natural low temperature treatment and then sampled after being returned to room temperature for 10 days. In this experiment, all samples were collected from young leaves of the plants and did not include the shoot apical meristem.

### CUT&Tag library preparation and sequencing

The CUT&Tag assay was performed according to a previously described protocol [37]. Approximately, 0.1 g of leaf tissue was used to isolate nuclei. The isolated nuclei were washed twice with wash buffer (20 mM HEPES, 150 mM NaCl, 0.5 mM spermidine and 0.1% protease inhibitor cocktail) and gently resuspended in 50 μL of antibody buffer (20 mM HEPES, 150 mM NaCl, 0.5 mM spermidine, 0.1% protease inhibitor cocktail, 2 mM EDTA and 0.1% BSA) containing the corresponding antibodies: H3K4me3 (abclonal, #A2357), H3K27ac (abclonal, #A2771), H3K27me3 (abclonal, #A16199), H3K36me3 (abclonal, #A20379), and H3K9me2 (abclonal, #A2359). After overnight incubation at 4°C, the primary antibody was removed by centrifugation, and the nuclei were incubated in 50 μL of antibody buffer with a secondary antibody for ~2 h at 4°C, followed by one wash with TIB buffer (20 mM HEPES, 300 mM NaCl, 0.5 mM spermidine and 0.1% protease inhibitor cocktail). A 1:100 dilution of pG-Tn5 complex (Vazyme, #S602) was prepared in TIB buffer and incubated with the nuclei for 1 h at 4°C. After incubation, the nuclei were washed twice with TIB buffer. Subsequently, the nuclei were resuspended in 300 μL of TIB buffer supplemented with 10 mM MgCl_2_ and incubated at 37°C for 1 h to allow tagmentation. To terminate the reaction, 10 μL of 0.5 M EDTA, 3 μL of 10% SDS and 4 μL of 10 mg/mL proteinase K were added, and the mixture was incubated at 50°C for 1 h. DNA was then extracted using phenol:chloroform:isoamyl alcohol (25:24:1), ethanol-precipitated, and resuspended in ddH_2_O. The library was amplified for 12-15 cycles using a DNA Library Prep Kit (Vazyme, #TD501) and purified with VAHTS DNA Clean Beads (Vazyme, #N411). All libraries were sequenced on the DNBSEQ-T7 system to generate 150 bp paired-end reads.

### ATAC-seq library construction and sequencing

ATAC-seq was performed following a previously described protocol [38] with slight modifications. Briefly, ~0.05 g of tissue was ground into a fine powder in liquid nitrogen and lysed in NIB buffer (20 mM HEPES pH 8.0, 250 mM sucrose, 1 mM MgCl_2_, 0.5 mM KCl, 40% glycerol, 0.25% Triton X-100, 0.1 mM phenylmethanesulfonyl fluoride (PMSF) and 0.1% 2-mercaptoethanol). The homogenate was filtered once through 40 μm Miracloth (Millipore, #475855). Nuclei were pelleted by centrifugation, washed with Tris-Mg buffer (10 mM Tris-HCl pH 8.0 and 5 mM MgCl_2_), and then resuspended in 40 μL of TTBL buffer (Vazyme, #TD501) containing Tn5 transposase. The reaction was incubated at 37°C for 30 min. DNA was extracted from the tagmented products using the CTAB method. Library amplification was carried out with the TruePrepTM DNA Library Prep Kit (Vazyme, #TD501) and purification was performed using VAHTS DNA Clean Beads (Vazyme, #N411). Libraries were sequenced on the DNBSEQ-T7 system to generate 150 bp paired-end reads.

### RNA-seq library construction and sequencing

The collected samples were immediately flash frozen in liquid nitrogen. Total RNA was extracted, and RNA-Seq libraries were constructed by Gene plus (Beijing, China). All libraries were sequenced on the DNBSEQ-T7 system to generate 150 bp paired-end reads.

### BL-Hi-C library preparation and sequencing

BL-HI-C libraries were constructed in accordance with established methods with some modifications. A key modification was the use of double-enzyme digestion (*Hpy*CH4V and *Alu*I) instead of the single-enzyme *(Hae*III) digestion used in the original protocol. Briefly, ~0.1 g of dual crosslinked rosette was ground in liquid nitrogen into fine powder, and subsequently lysed with NIB (20 mM Hepes (pH 8), 250 mM sucrose, 1 mM MgCl_2_, 0.5 mM KCl, 40% glycerol, 0.25% Triton X-100, 0.1 mM PMSF and 0.1% 2-mercaptoethanol). The slurry containing the nuclei extract was filtered once through 40 μm Miracloth (Millipore, #475855). Subsequently, the nuclei were washed twice with 1.3× CutSmart Buffer (NEB, #B6004). After nuclei extraction, it was subjected to enzymatic digestion with *Alu*I (NEB, #R0137S) and *Hpy*CH4V (NEB, #R0620L) for 12 h. The digested DNA was then ligated to linkers using T4 ligase (NEB, #M0202L). After reverse crosslinking, the proximity-ligated DNA was extracted, and prepared for BL-Hi-C library construction (VAHTS, #ND608). The libraries were sequenced using the DNBSEQ-T7platform (paired-end 150 bp).

### Processing of RNA-seq data

For quality control, raw reads were processed with Trimmomatic v0.39 [39]. Clean reads were then aligned to the *B. rapa* reference genome (Brapa_chiifu_V3.5) [40] using HISAT2 (https://daehwankimlab.github.io/hisat2/) with default parameters. The resulting BAM files were sorted using SAMtools v1.1.4 [41]. Gene expression read counts were obtained with featureCounts [42], and fragments per kilobase per million (FPKM) values were calculated using custom scripts.

### Processing of ATAC-seq data

For data quality control, raw reads were processed using Trimmomatic v0.39 [39]. Clean reads were aligned to the *B. rapa* reference genome (Brapa_chiifu_V3.5) [40] with Bowtie2 v2.4.2 [43] under default parameters. SAMtools v1.1.4 [41] was used to sort BAM files, filter alignments (-q 10), and remove duplicate reads. Peak calling was performed using MACS2 v2.2.7.1 [44] with the following parameters for ATAC-seq data: macs2callpeak-g 351 000 000 --shift -100 --extsize 200 --nomodel.

### Processing of CUT&Tag data

Data quality control and alignment procedures followed the workflow used for ATAC-seq analysis. Peaks calling for CUT&Tag data of H3K4me3, H3K27ac and H3K9me2, the following parameters were used: macs2 callpeak -g 351 000 000 --extsize 200 --nomodel. Peaks calling for H3K27me3 and H3K36me3 data, the --broad flag was included: macs2 callpeak -g 351 000 000 --extsize 200 --nomodel --broad.

### Processing of BL-Hi-C data

The trimLinker of ChIA-PET2 (v0.9.3) was used to filter the linker (linker: CGCGATATCTTATCTGACT). Reads were aligned to the *B. rapa* reference genome (Brapa_chiifu_V3.5) [40] using bwa v0.7.17 with single mode. SAMtools v1.1.4 was used to convert sam to bam (-bS -@ 10 -h -F 2048). HiC-Pro v3.1.0 was used to remove duplicates, perform quality control, and build contact matrix (-s proc_hic -s merge_persample -s build_contact_maps). Compartments were identified using Juicer v1.9.9 at 20 Kb resolution.

### Dual-luciferase assay

The genomic sequence of distal CREs were amplified and fused in frame with the pGreenII-0800-miniLUC vector to generate the reporter construct CREs-mini35Spro:LUC. Then, mini35Spro:LUC (as control) and the reporter vector CREs-mini35Spro:LUC were transformed into *Agrobacterium tumefaciens* strain GV3101. The bacterial solution was injected to the back of the leaves of *N. benthamiana* (6-8 leaf stage) using a syringe with the needle removed. The *N. benthamiana* were cultivated for 2-3 days at a temperature of 25°C and a light cycle of 16 h light/8 h dark. Luciferase activity was quantified using reagents from the Dual Luciferase Reporter Gene Assay Kit (Yeasen, 11402ES60) according to the manufacturer’s instructions. The relative intensity was calculated by ratio between relative ratio (LUC: REN) of CREs and empty vector. The primer sequences are listed in Table S6.

## Supplementary Material

Web_Material_uhag236

## Data Availability

Raw sequencing data generated in the course of this study have been deposited to the National Genomics Data Center (NGDC) database under accession number PRJCA051260.
