# Supplementary material for: Distal cis-regulatory elements in 3′ UTRs orchestrate gene expression via single-gene domain architecture in Brassica rapa
Source: Hortic Res. 2026 Jun 10;13(11):uhag236. doi: 10.1093/hr/uhag236 (PMC13735537; doi:10.1093/hr/uhag236)
Supplement: Supplementary file 1 [file web_material_uhag236.zip › Supplement_figure-20260521.docx]

Supplementary

**Figure S1** Representative images of *B. rapa* acc. Chiifu at the rosette and reproductive stages. The red arrow indicates the flower buds of *B. rapa* at the reproductive stage.

**Figure S2**. Correlation analysis of reads intensity between replicates for ATAC-seq and Cut&Tag (H3K3me3, H3K27ac, H3K27me3, H3K36me3 and H3K9me2). The reads number per 10kb was used for read intensity evaluation. Pearson's correlation coeficients (R) are shown at the upper.

**Figure S3**. Quality control of BL-Hi-C data. Heatmap showing reproducibility between biological replicates at rosette and reproductive stages, analyzed using the estSim module of cLoops2 (Cao et al. 2022) at 500-bp resolution.

**Figure. S4** A sets of representative example shows the consistent single-gene domain structures in two biological replicates.

**Figure S5.** Chromatin states of *B. rapa* leaf at reproductive stage.

**Figure S6.** Similar domain structure of the *BrKAN2* in both rosette and reproductive stages. In *B. rapa*, *BrKAN2* has two copies, namely *BrKAN2.1* (*BraA09g034220.3.5C*) and *BrKAN2.3* (*BraA05g024090.3.5C*).

**Figure S7**. Reduced domain structure of the *BrFLC1* (*BraA10g027790.3.5C*) and *BrFLC3* (*BraA03g004250.3.5C*) genes from the rosette to reproductive stages.

**Cao Y, Liu S, Ren G, Tang Q, and Zhao K**. cLoops2: a full-stack comprehensive analytical tool for chromatin interactions. Nucleic Acids Research. 2022:**50**(1):57–71. https://doi.org/10.1093/nar/gkab1233
